# Supplementary material for: Genetic circuit characterization and debugging using RNA‐seq
Source: Mol Syst Biol. 2017 Nov 9;13(11):952. doi: 10.15252/msb.20167461 (PMC5731345; doi:10.15252/msb.20167461)
Supplement: Supplementary file 1 — Appendix [file MSB-13-952-s001.pdf]

Appendix for:

Genetic circuit characterization and debugging using RNA-seq

Thomas E. Gorochowski, Amin Espah Borujeni, Yongjin Park, Alec A.K. Nielsen, Jing Zhang, Bryan S. Der, D. Benjamin Gordon and Christopher A. Voigt

**Appendix Text**		**2**
Appendix Text S1:	Transcription profile correction method	2
Appendix Text S2:	Measurement of ribozyme performance from RNA-seq data	5
**Appendix Figures**		**6**
Appendix Figure S1:	Distribution of fragment length versus circuit position	6
Appendix Figure S2:	Transcription profile replicates, measured on different days	7
Appendix Figure S3:	Sequenced fragment length distributions	8
Appendix Figure S4:	Ribozyme transcription profiles for cells grown in culture tubes	9
Appendix Figure S5:	Antisense transcription across the circuit	10
Appendix Figure S6:	Transcription profiles for parts when cells are grown in Erlenmeyer flasks	11
Appendix Figure S7:	Sensor and gate response functions when cells are grown in Erlenmeyer flasks	12
Appendix Figure S8:	Circuit plasmid	13
**Appendix Tables**		**14**
Appendix Table S1:	Ribozyme part characterization in culture tubes	14
Appendix Table S2:	Top 25 down and up regulated genes for states where four circuit genes are expressed	15
Appendix Table S3:	Promoter and terminator part characterization in Erlenmeyer flasks	16
Appendix Table S4:	Sensor and gate response function parameters in Erlenmeyer flasks	17
Appendix Table S5:	Genetic part sequences	18
**Appendix References**		**21**

Appendix Text S1: Transcription profile correction method

RNA-seq measurements generate millions of fragments that are computationally mapped to sequences for the genetic circuit and host genome. The transcription profile is created via this process. This is adequate for many applications in systems biology where gene expression can be inferred by averaging the profile across the length of a gene. However, there is a bias that occurs during the mapping process that causes a gradual decline at the 5'- and 3'- ends of each transcription unit. By definition, promoters and terminators occur at these boundaries. While it is easy to qualitatively detect that these parts exist (and this is the basis for algorithms to detect promoters/terminators in the genome), the decline complicates the quantitative calculation of their strength, which is important in synthetic biology. Therefore, we developed a method to correct for these edge effects that utilizes the empirical fragment length distribution from the RNA-seq experiment. The distribution is used to create a single correction factor that is applied to all of the 5'- and 3'- ends of the transcripts in the circuit. After this process, the equations to calculate part strength are applied. Note that this correction factor is only applied to the transcripts corresponding to the gates in the circuit; it is not applied to internal promoters, antisense transcription, or genomic expression (in these cases, it is not necessary to calculate part strength). Below, when we refer to the transcripts in the circuit, this only encompasses transcripts originating from transcription start sites within the input/output promoters in the gates.

Each RNA-seq experiment has a unique fragment length distribution, which depends on many growth-related and growth-unrelated processes (Klumpp et al, 2009; Roberts et al, 2011). Inside cells, the length and abundance of mRNA transcripts at steady-state is controlled by a balance between several processes such as transcriptional bursting, elongation and termination, RNA polymerase fall-off, and mRNA degradation. Outside the cells, after total RNA extraction, mRNA transcripts undergo several post-processing modifications such as fragmentation, reverse transcription, PCR amplification, and DNA selection before being sequenced using high-throughput sequencing systems. Importantly, these post-processing steps will define the final shape of fragment length distribution. Two main contributing factors to this shape are the positional biases introduced during the post-processing and sequencing events, and the loss of RNA fragments (mostly occurring during the DNA selection step).

The profile bias at the ends of a transcript occurs because a fragment is more likely to map at the center of the transcript and less likely at the ends. Differences in the fragment length distribution impact the shape of the profile. Long fragments have the effect of exaggerating the bias (it extends deeper into the transcript from each end) as compared to short ones. Here, we sought to create a simple method that calculates a correction factor based on the fragment length distribution and apply it to all the transcription

units in the circuit. Alternatively, the fragment length distribution for each transcription unit, as opposed to the experiment as a whole, could be calculated and applied. However, we found that this was unnecessary because the transcription units are all about the same size due to the fact that they encode single TetR-family repressors.

The first step in our correction method is to generate a distribution of all the fragment lengths mapped across the circuit and the genome (Appendix Figure S3). Because we use paired-end sequencing, the position of the start and end of each fragment is known, enabling the length to be directly calculated. We then consider a hypothetical 2000 nt transcription unit to generate a calculated profile *T(x)* that captures the expected curvature across a transcript within the transcription profile. This is generated by stochastically selecting a number of lengths (100,000) from the fragment length distribution and randomly mapping fragments of these lengths to the hypothetical transcription unit. *T(x)* is then produced by counting the number of mapped fragments per nucleotide (Box 1). In order to generate a general correction factor profile *C(x)*, the hypothetical profile *T(x)* is normalized with respect to its maximum. We found that the impact of the correction is negligible after ~400 nt , in other words $C(x) \longrightarrow 1$, so only the first 500 nt of *C(x)* are used. This correction factor is only based on the length of the fragments and does not consider their sequence. Thus, the effect is identical at the 5'- and 3'- ends of the transcript and the same correction factor can be applied to both ends.

Next, the transcription profile *P(x)* is generated for transcription units across the circuit. It is not applied to regions where part strengths do not have to be calculated, such as genomically-encoded genes. First, fragments from the RNA-seq data mapping within the boundaries of a single transcription unit are identified. These are used to generate *P(x)* by counting the number of fragments covering each nucleotide *x*. To correct the curvature present at the ends of each transcription unit (Box 1), we divide the first and last 500 nt of each transcription unit in *P(x)* by $C(x_n)$, where x_n is the distance in nucleotides to the nearest end of the transcription unit. Finally, all remaining fragments from the RNA-seq data (*i.e.*, those mapping outside the previously considered transcription units or spanning transcription unit boundaries) are combined with $P_c(x)$ to produce the final transcription profile. To enable comparison of absolute changes in the profiles between samples, *M(x)* is further normalized by $F = m/10^6$, where *m* is the effective library size calculated for each RNA-seq experiment using a trimmed mean of M-values (TMM) approach (Robinson & Oshlack, 2010) (Dataset EV1).

This method accurately corrects the curvature at the two ends of all transcription units in the circuit. However, issues were observed for the *LitR* and *BM3R1* genes for state –/–/+ when grown in culture tubes (specifically for biological replicate 1). For these genes, the 3'-end of the corrected profiles unexpectedly

rises (Figure 2D). This was due to a large number of fragments having their 3’-end mapping to the 3’-end of these transcription units (Appendix Figure S1). This feature was only present for one of the biological replicates and not seen across the other samples (Appendix Figure S2).

<u>Appendix Text S2:</u> Measurement of ribozyme performance from RNA-seq data

Ribozyme insulators were present at all promoter-RBS junctions to cleave variable 5' sequences generated by differing upstream promoters. Inspection of the transcription profiles revealed that for both single and pairs of promoters, increases in the profiles occurred not at the transcription start site (x_{TSS}) of each promoter, but instead at the cut-site of the nearest downstream ribozyme insulator (Appendix Figure S5B). The reason for this could be traced to the preparation of the sequencing libraries. Because ribozymes are located near the start of the 5'-UTR of each transcript, after cleavage, a short ~80 bp fragment is generated in addition to a longer fragment containing the downstream gene. Short-cleaved fragments were undetected during sequencing because reverse transcribed cDNA fragments of less than 100 bp were filtered during library preparation (Appendix Figure S4). This resulted in the transcription profiles lacking information for the beginning of these transcripts.

A byproduct of this filtering was that it allowed us to characterize ribozyme cleavage. Because short cleaved RNA fragments were filtered and uncleaved fragments were captured during sequencing, by comparing the transcription profile directly after the cut-site (capturing both cleaved and uncleaved fragments) to the transcription profile at the beginning of the ribozyme (only capturing uncleaved fragments), the fraction of cleaved fragments p_c can be calculated as

$$p_C = \frac{\sum_{i=x_C+1}^{x_C+n} M(i) - \sum_{i=x_0-1}^{x_0-n} M(i)}{\sum_{i=x_C-1}^{x_C-n} M(i) - \sum_{i=x_0-1}^{x_0-n} M(i)}. \tag{S1}$$

Here, x_C is the position of the ribozyme's cut-site, x_0 is the start position of the first upstream promoter, and n is the window length (Appendix Figure S5A). Transcripts originating from upstream of the ribozyme's associated promoter (Appendix Figure S5A) are subtracted because cleavage of these will generate fragments with a length that is too large to be filtered during library preparation and would thus confound the calculation.

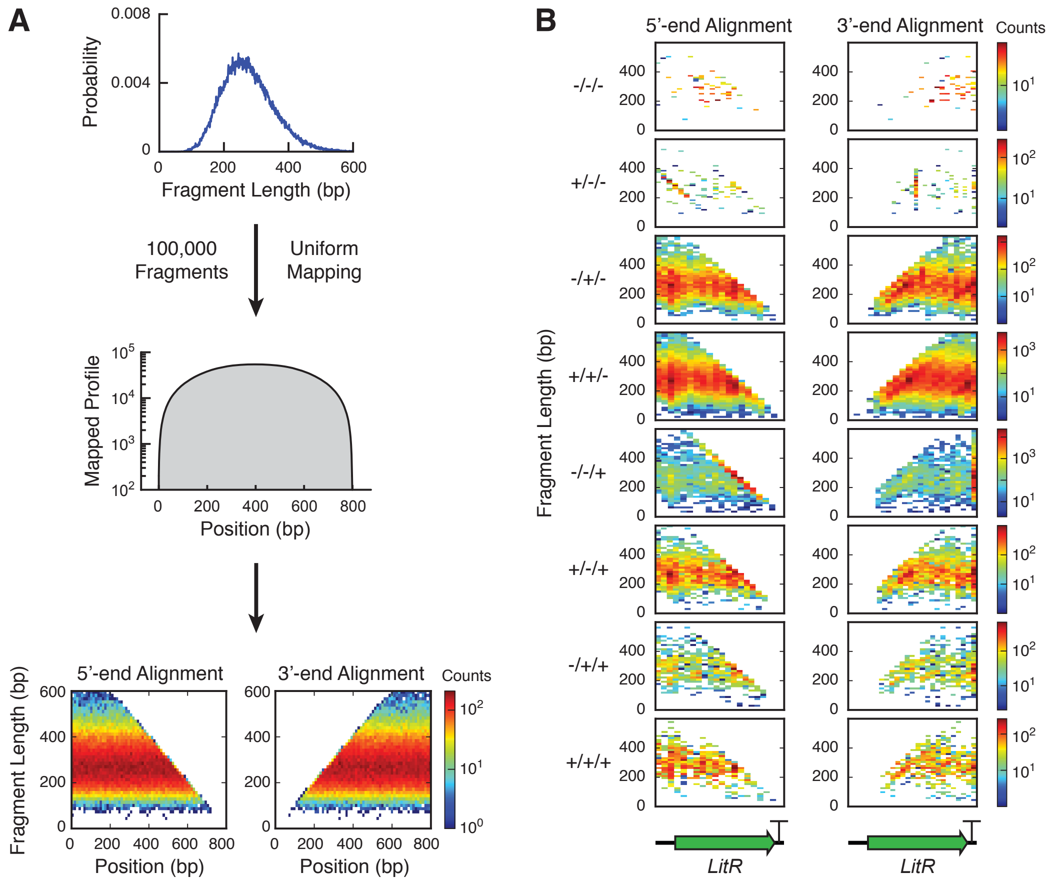

<u>Appendix Figure S1:</u> Distribution of fragment length versus circuit position. (**A**) A hypothetical fragment length distribution was generated by randomly selecting 100,000 values from a gamma probability density function that mimics the experimental fragment length distributions (shape = 12 and scale = 23). Fragments were *uniformly* mapped to random positions within an 800 nt hypothetical transcription unit, and a transcription profile generated by counting the number of fragments spanning each nucleotide position. Heat-maps show how fragments of different lengths are distributed across the transcription unit. At all positions, the number of mapped fragments with specific lengths follows the original fragment length distribution. The right-angled trapezoid shape of the heat-maps is the result of fragments having to map within the boundaries of the transcription unit. (**B**) Distribution of length versus position are shown for fragments mapped exclusively within the borders of *LitR*. Data shown for experiments performed in culture tubes. *LitR* is actively transcribed for six induction states (–/+/–, +/+/–, –/–/+, +/–/+, –/+/+ and +/+/+). These states show a near uniform mapping and no positional bias, apart from state –/–/+. For this state, a significant number of fragments map to the 3'-end of *LitR*, which explains the rise in the corrected transcription profiles at this point (Figure 2D).

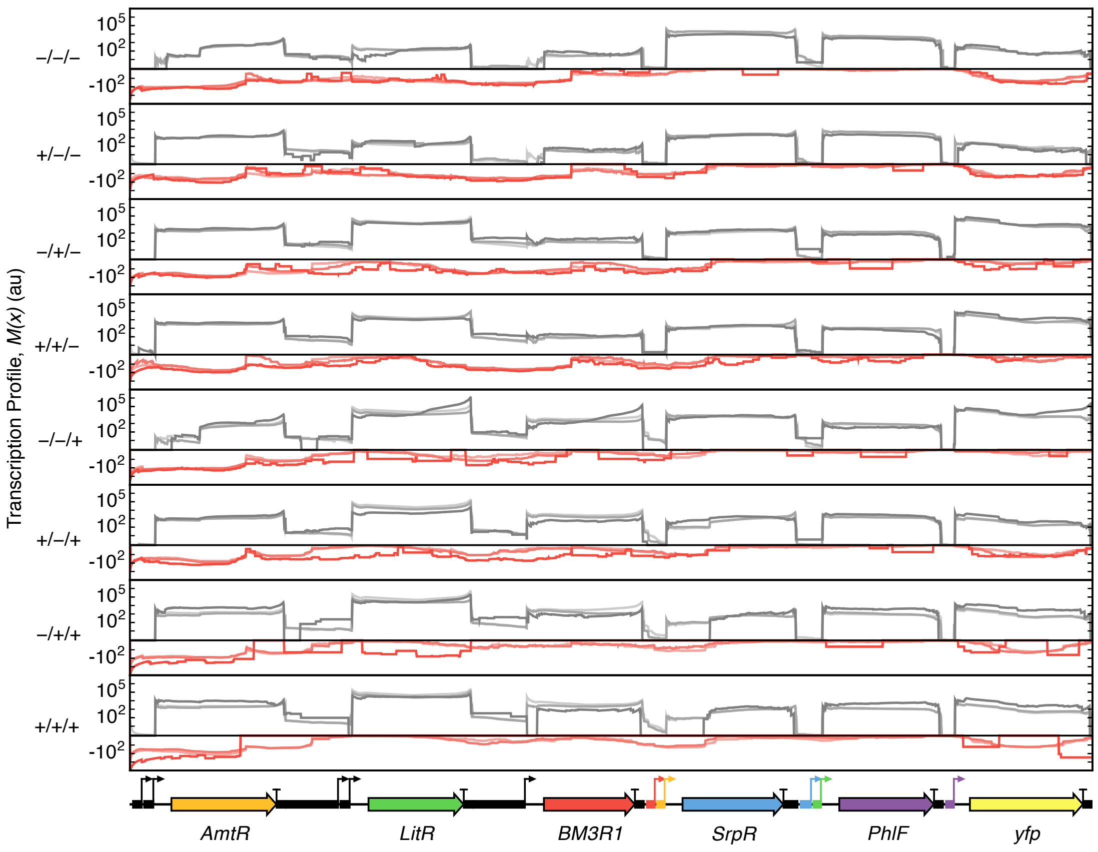

<u>Appendix Figure S2:</u> Transcription profile replicates, measured on different days. Separate lines are shown for each of the three biological replicates. Transcription profiles for the sense strand are colored grey and red for the antisense strand.

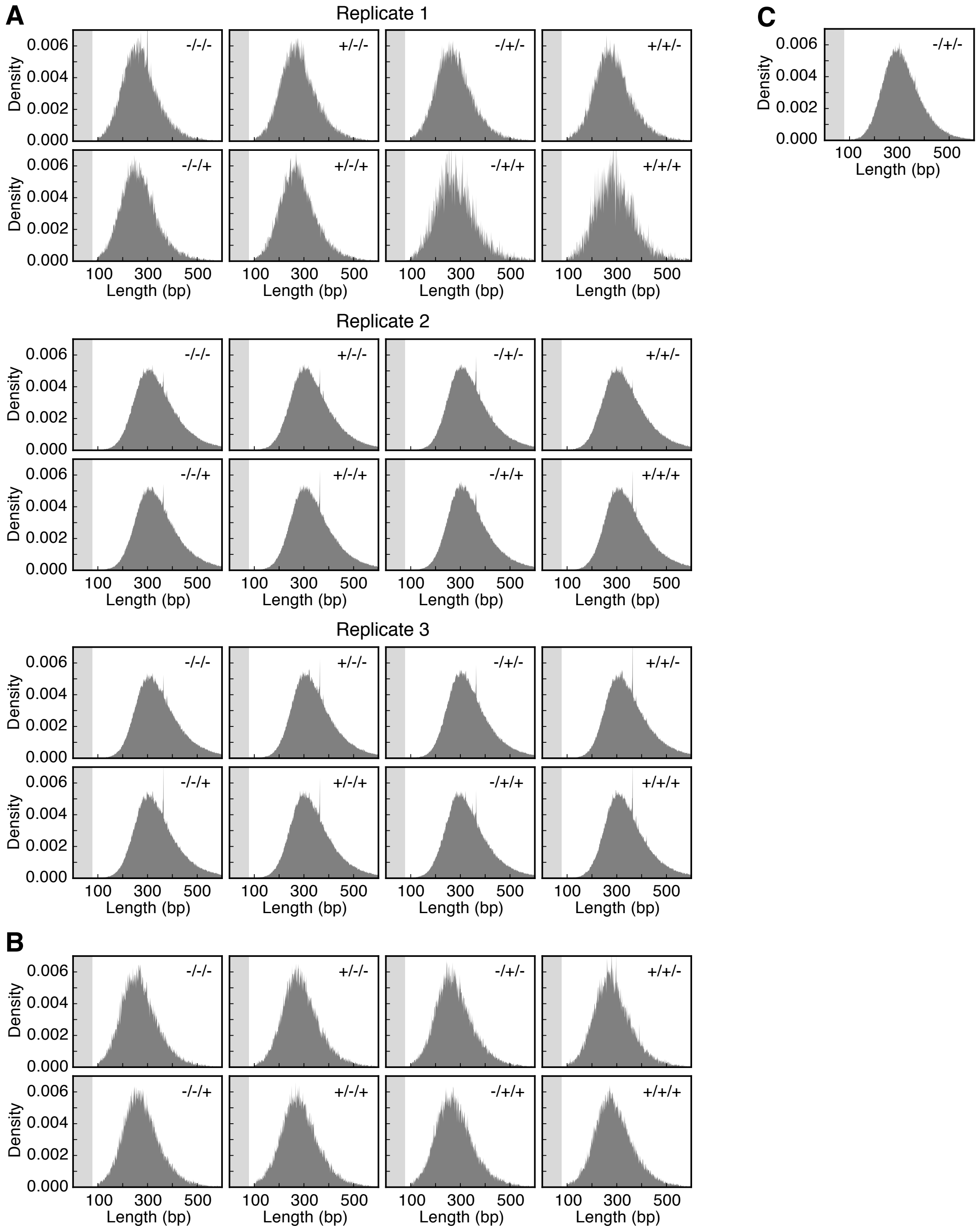

<u>Appendix Figure S3:</u> Sequenced fragment length distributions. (**A**) Circuit grown in culture tubes (data shown for three biological replicates) and (**B**) Erlenmeyer flasks for all combinations of inputs. (**C**) Modified version of the circuit grown in culture tubes. Each distribution shows the combination of inputs (IPTG/aTc/Ara) in the top right corner. The shaded regions (0 to 78 bp) represent the length of the longest 5'-UTR fragment produced after ribozyme cleavage.

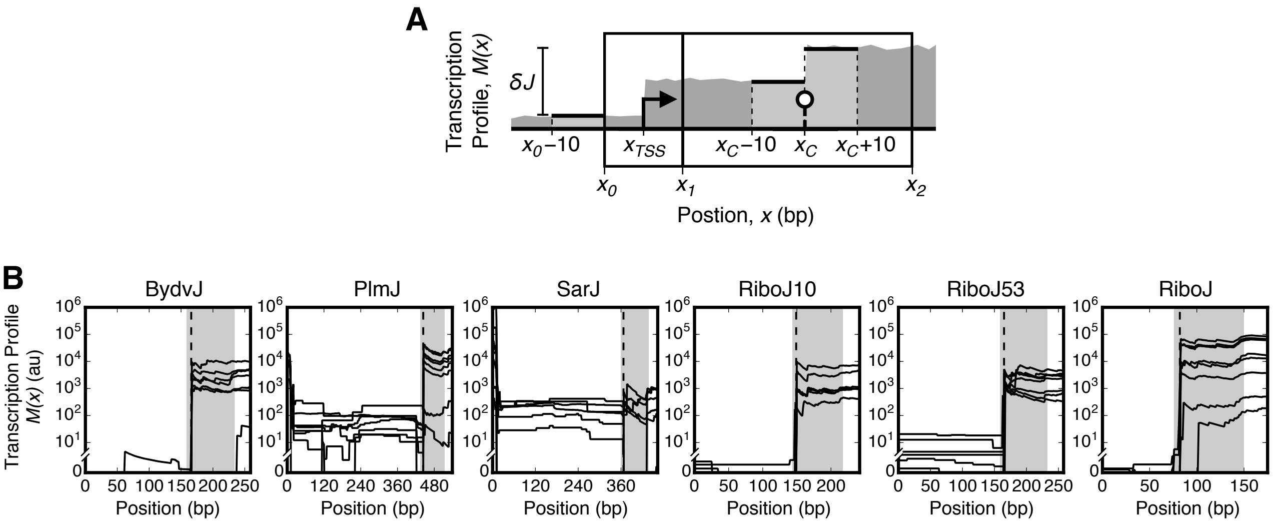

<u>Appendix Figure S4:</u> Ribozyme transcription profiles for cells grown in culture tubes. (**A**) Method for characterizing ribozyme cleavage (Appendix Text S2). The fraction of cleaved fragments by the ribozyme is calculated as $p_c = \left[\sum_{i=x_C+1}^{x_C+n} M(i) - \sum_{i=x_0-1}^{x_0-n} M(i)\right] / \left[\sum_{i=x_C-1}^{x_C-n} M(i) - \sum_{i=x_0-1}^{x_0-n} M(i)\right]$ and the activity of the associated promoter is given by $\delta J = \frac{\gamma}{n}\left[\sum_{i=x_C+1}^{x_C+n} M(i) - \sum_{i=x_0-1}^{x_0-n} M(i)\right]$ with n = 10 bp. (**B**) Transcription profiles for the ribozyme parts. Lines show the transcription profile for each of the 8 input states. Shaded regions denote the location of the ribozyme and dashed line shows the cleavage site. Data shown for biological replicate 1.

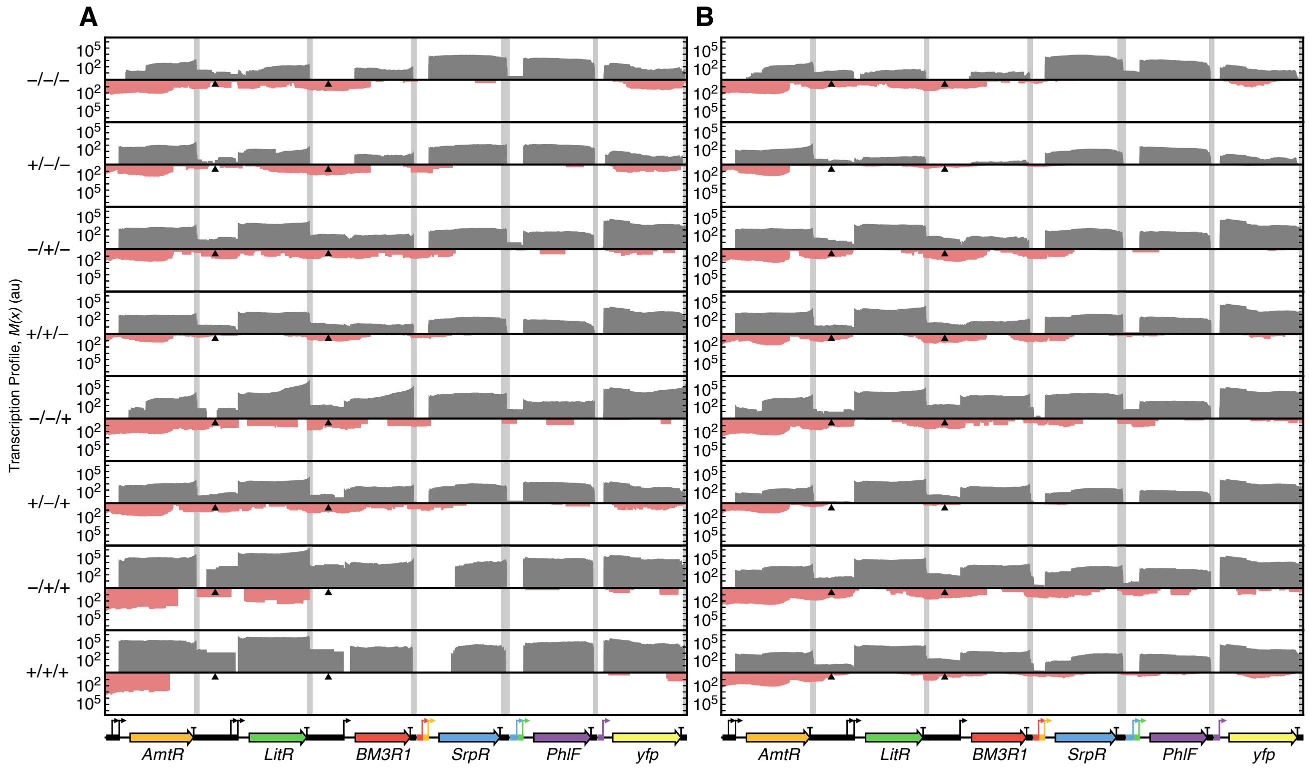

<u>Appendix Figure S5:</u> Antisense transcription across the circuit. Data for all input states shown for cells grown under (**A**) culture tube (data shown for biological replicate 1) and (**B**) Erlenmeyer flask conditions. Transcription profiles are shown for both sense (gray) and antisense (red) strands. Light gray shaded regions denote the location of terminator parts. Triangles mark the antisense promoter present within P_{BAD}.

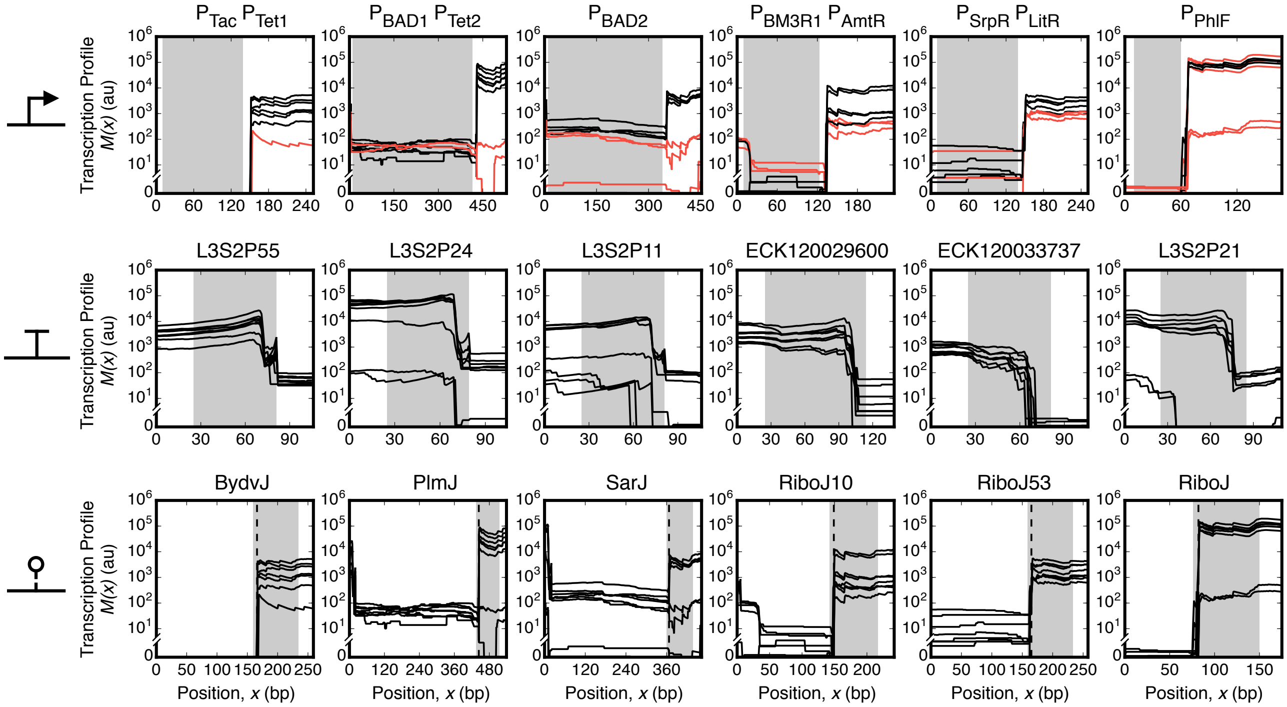

<u>Appendix Figure S6:</u> Transcription profiles for parts when cells are grown in Erlenmeyer flasks. Lines show the transcription profile for each of the 8 input states. Shaded regions denote the location of the relevant part. For promoters, the lines are black when the promoter is expected to be on and red when it is expected to be off. The ribozyme cleavage site is denoted by a dashed line.

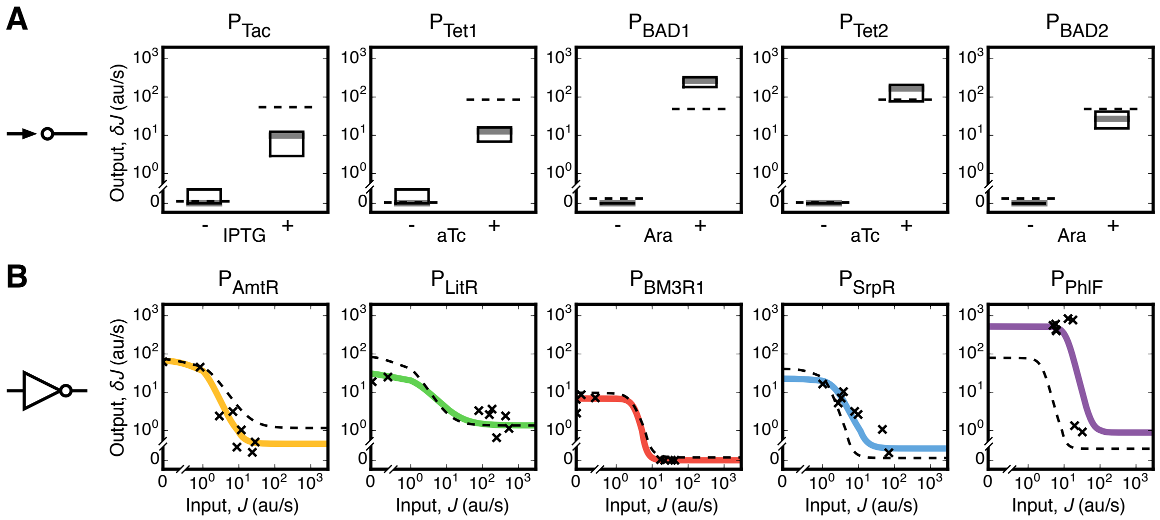

Appendix Figure S7: Sensor and gate response functions when cells are grown in Erlenmeyer flasks. (**A**) The response of the output promoters of the sensors are shown in the presence and absence of each inducer. The dashed lines show the sensor outputs measured in isolation (Nielsen et al, 2016). The boxes show the median (grey line) and range of promoter activities measured for the four states where it is off (δJ_{off}) and four where it is on (δJ_{on}). (**B**) Solid colored lines show the response functions of the gates obtained by fitting the promoter activities to the RNA-seq data (circles denote the measured values for the 8 input states). The dashed lines show the output of the gate measured in isolation (Nielsen et al, 2016). The fit parameters for the response functions are provided in Appendix Table S4.

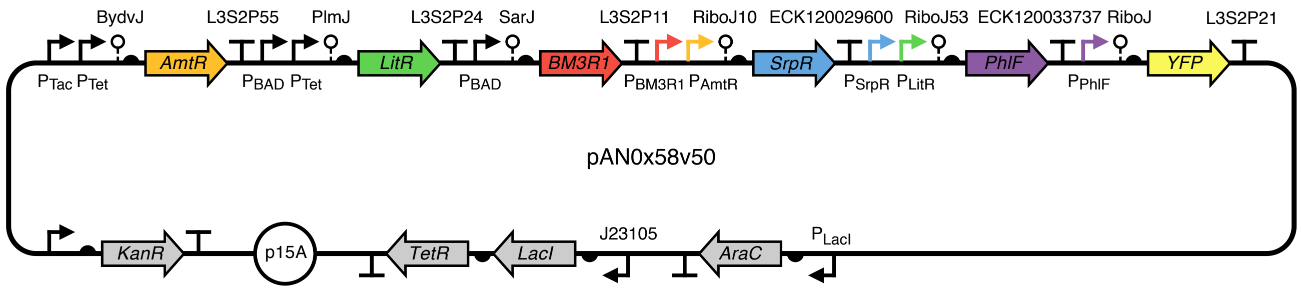

Appendix Figure S8: Circuit plasmid. Parts names are shown for all genes, promoters, terminators, and ribozymes. Part sequences are provided in Appendix Table S5.

Appendix Table S1: Ribozyme part characterization in culture tubes.

Ribozyme	Genetic Context	
	Isolation[a,b]	Circuit[b,c]
BydvJ	0.98	0.99 ± 0.0
PlmJ	0.97	0.99 ± 0.0
SarJ	0.99	0.99 ± 0.0
RiboJ10	0.98	0.99 ± 0.0
RiboJ53	0.99	0.98 ± 0.0
RiboJ	0.97	0.99 ± 0.04

a. Measurements from Nielsen et al (2016).

b. Ribozyme cleavage values given as a fraction of the total cleaved to 2 s.f.

c. Average and standard deviation calculated from three replicates performed on different days for states where the transcript is expressed.

Appendix Table S2: Top 25 down and up regulated genes for states where four circuit genes are expressed

Gene	log_2 fold-change	Description	Related pathways and role
yncA	-5.0	50S ribosomal protein L36 paralog	Protein acetylation
flu	-3.6	Self-recognizing antigen 43 (Ag43) autotransporter	Integral component of membrane
ygiP	-3.6	DNA-binding transcriptional activator TtdR	–
yqeC	-3.8	Uncharacterized protein	–
ydcZ	-3.3	Uncharacterized protein	–
napF	-4.2	Ferredoxin-type protein	Response to oxidative stress
ndk	-2.4	Nucleoside diphosphate kinase	Nucleotide biosynthesis
nikA	-3.4	Ni(2+) ABC transporter periplasmic binding protein	Nickel cation transport
dmsA	-3.8	Dimethyl sulfoxide reductase subunit A	Anaerobic respiration
tpx	-2.0	Lipid hydroperoxide peroxidase	Cellular response to oxidative stress
rraA	-2.8	Ribonuclease E inhibitor protein A	RNA metabolism
ansB	-2.7	Asparaginase II	Amino acid metabolism
yjjI	-3.8	Uncharacterized protein	–
yedE	-3.3	Uncharacterized protein	–
hypB	-3.1	GTP hydrolase (nickel liganding into hydrogenases)	Protein maturation and complex assembly
yccM	-3.0	Uncharacterized protein	–
napA	-3.6	Periplasmic nitrate reductase subunit	Anaerobic respiration
yhbU	-3.5	Uncharacterized protein	–
yjjW	-3.2	Uncharacterized protein	–
yhjX	-2.5	Uncharacterized protein	–
sodB	-1.6	Superoxide dismutase (Fe)	Oxidation-reduction process
csiE	-2.4	Stationary phase inducible protein	Transcription regulation
pepT	-2.4	Peptidase T	Proteolysis
yiaU	-2.4	Uncharacterized protein	–
yncA	-5.0	L-amino acid N-acyltransferase	Protein acetylation
nrdE	3.6	Ribonucleoside-diphosphate reductase 2, α subunit dimer	DNA replication
nrdF	3.7	Ribonucleoside-diphosphate reductase 2, β subunit dimer	DNA replication
nrdI	4.1	Flavodoxin	Protein modification
yjjZ	4.3	Uncharacterized protein	–
rcsA	5.8	DNA-binding transcriptional activator	Transcriptional regulation of colonic acid
fhuE	3.0	Ferric coprogen/ferric rhodotorulic acid transporter	Iron ion homeostasis
yedA	3.4	Uncharacterized protein	–
sufD	2.0	Fe-S cluster scaffold complex subunit	Response to oxidative stress
nrdH	3.1	Glutaredoxin-like protein	Cell redox homeostasis
sufS	2.0	L-cysteine desulfurase	Sulphur compound metabolism
yjbE	5.9	Uncharacterized protein	–
add	1.8	Adenosine deaminase	DNA damage response and purine salvaging
xylF	4.8	Xylose ABC transporter periplasmic binding protein	Carbohydrate transport
fhuF	1.5	Hydroxamate siderophore iron reductase	Iron assimilation
araJ	4.6	Uncharacterized protein	–
eptA	2.5	Phosphoethanolamine transferase	Response to antibiotics and lipid metabolism
acrD	2.1	Multidrug efflux pump RND permease	Response to drug and drug transport
yagG	1.6	Uncharacterized protein	–
asnB	1.7	Asparagine synthetase B	Amino acid biosynthesis
yedV	2.2	Sensory histidine kinase	DNA damage response
cirA	2.0	Ferric dihyroxybenzoylserine outer membrane transporter	Iron assimilation
sufC	2.0	Fe-S cluster scaffold complex subunit	Iron-sulfur cluster assembly
ydeH	2.1	Diguanylate cyclase	Regulation of cell motility
gmd	4.9	GDP-mannose 4,6-dehydratase	Colanic acid biosynthesis
sufE	2.1	Sulfur acceptor for SufS cysteine desulfurase	Response to oxidative stress

Appendix Table S3: Promoter and terminator part characterization in Erlenmeyer flasks.

Promoter(s)	Strength[a]
P_{Tac}-P_{Tet1}	6, 9
P_{BAD1}-P_{Tet2}	213, 115
P_{BAD2}	27
P_{BM3R1}-P_{AmtR}	7, 45
P_{SrpR}-P_{LitR}	12, 26
P_{PhlF}	533
**Terminator**	**Strength[b]**
L3S2P55	25
L3S2P24	179
L3S2P11	70
ECK120029600	260
ECK120033737	793
L3S2P21	98

a. Average promoter strengths are shown in au/s for each promoter when on. For double promoters, strengths are calculated separately when only one of the promoters is predicted to be on.

b. Median terminator strengths are calculated for states where the upstream gene is predicted to be in an on state.

Appendix Table S4: Sensor and gate response function parameters in Erlenmeyer flasks.

Sensor[a]	δJ_{off}	δJ_{on}		
P_{Tac}	0.0	10		
P_{Tet1}	0.0	13		
P_{Tet2}	0.0	167		
P_{BAD1}	0.0	265		
P_{BAD2}	0.0	27		
**Gate**[b]	δJ_{out}^{min}	δJ_{out}^{max}	K	n
P_{AmtR}	0.6	66	1.1	2.3
P_{LitR}	1.4	31	1.6	1.3
P_{BM3R1}	0.0	7	3.3	4.0
P_{SrpR}	0.4	23	2.4	2.3
P_{PhlF}	0.9	523	11.6	4.0

a. In units of au/s.

b. Parameters δJ_{out}^{min}, δJ_{out}^{max} and K are in units au/s.

Appendix Table S5: Genetic part sequences

Part Name	Type	DNA Sequence
AmtR	Gene	ATGGCAGGCGCAGTTGGTCGTCCGCGTCGTAGTGCACCGCGTCGTGCAGGTAAAAATCCGCGTGAA<br>GAAATTCTGGATGCAAGCGCAGAACTGTTTACCCGTCAGGGTTTTGCAACCACCAGTACCCATCAG<br>ATTGCAGATGCAGTTGGTATTCGTCAGGCAAGCCTGTATTATCATTTTCCGAGCAAAACCGAAATC<br>TTTCTGACCCTGCTGAAAAGCACCGTTGAACCGAGCACCGTTCTGGCAGAAGATCTGAGCACCCTG<br>GATGCAGGTCCGGAAATGCGTCTGTGGGCAATTGTTGCAAGCGAAGTTCGTCTGCTGCTGAGCACC<br>AAATGGAATGTTGGTCGTCTGTATCAGCTGCCGATTGTTGGTAGCGAAGAATTTGCAGAATATCAT<br>AGCCAGCGTGAAGCACTGACCAATGTTTTTCGTGATCTGGCAACCGAAATTGTTGGTGATGATCCG<br>CGTGCAGAACTGCCGTTTCATATTACCATGAGCGTTATTGAAATGCGTCGCAATGATGGTAAAATT<br>CCGAGTCCGCTGAGCGCAGATAGCCTGCCGGAAACCGCAATTATGCTGGCAGATGCAAGCCTGGCA<br>GTTCTGGGTGCACCGCTGCCTGCAGATCGTGTTGAAAAAACCCTGGAACTGATTAAACAGGCAGAT<br>GCAAAATAA
LitR	Gene	ATGGATACCATTCAGAAACGTCCGCGTACCCGTCTGAGTCCGGAAAAACGTAAAGAACAGCTGCTG<br>GATATTGCCATTGAAGTTTTTAGCCAGCGTGGTATTGGTCGTGGTGGTCATGCAGATATTGCAGAA<br>ATTGCACAGGTTAGCGTTGCAACCGTGTTTAACTATTTTCCGACCCGTGAAGATCTGGTTGATGAT<br>GTTCTGAACAAAGTGGAAAACGAGTTTCACCAGTTCATCAATAACAGCATTAGCCTGGATCTGGAT<br>GTTCGTAGCAATCTGAATACCCTGCTGCTGAACATTATTGATAGCGTTCAGACCGGCAACAAATGG<br>ATTAAAGTTTGGTTTGAATGGTCAACCAGCACCCGTGATGAAGTTTGGCCTCTGTTTCTGAGCACC<br>CATAGCAATACCAATCAGGTGATCAAAACCATGTTTGAAGAGGGTATTGAACGCAATGAAGTGTGC<br>AATGATCATACACCGGAAAATCTGACCAAAATGCTGCATGGTATTTGCTATAGCGTGTTTATTCAG<br>GCCAATCGTAATAGCAGCAGCGAAGAAATGGAAGAAACCGCAAATTGCTTTCTGAATATGCTGTGC<br>ATCTACAAATAA
BM3R1	Gene	ATGGAAAGCACCCCGACCAAACAGAAAGCAATTTTTAGCGCAAGCCTGCTGCTGTTTGCAGAACGT<br>GGTTTTGATGCAACCACCATGCCGATGATTGCAGAAAATGCAAAAGTTGGTGCAGGCACCATTTAT<br>CGCTATTTCAAAAACAAAGAAAGCCTGGTGAACGAACTGTTTCAGCAGCATGTTAATGAATTTCTG<br>CAGTGTATTGAAAGCGGTCTGGCAAATGAACGTGATGGTTATCGTGATGGCTTTCATCACATTTTT<br>GAAGGTATGGTGACCTTTACCAAAAATCATCCGCGTGCACTGGGTTTTATCAAAACCCATAGCCAG<br>GGCACCTTTCTGACCGAAGAAAGCCGTCTGGCATATCAGAAACTGGTTGAATTTGTGTGCACCTTT<br>TTTCGTGAAGGTCAGAAACAGGGTGTGATTCGTAATCTGCCGGAAAATGCACTGATTGCAATTCTG<br>TTTGGCAGCTTTATGGAAGTGTATGAAATGATCGAGAACGATTATCTGAGCCTGACCGATGAACTG<br>CTGACCGGTGTTGAAGAAAGCCTGTGGGCAGCACTGAGCCGTCAGAGCTAA
SrpR	Gene	ATGGCACGTAAAACCGCAGCAGAAGCAGAAGAAACCCGTCAGCGTATTATTGATGCAGCACTGGAA<br>GTTTTTGTTGCACAGGGTGTTAGTGATGCAACCCTGGATCAGATTGCACGTAAAGCCGGTGTTACC<br>CGTGGTGCAGTTTATTGGCATTTTAATGGTAAACTGGAAGTTCTGCAGGCAGTTCTGGCAAGCCGT<br>CAGCATCCGCTGGAACTGGATTTTACACCGGATCTGGGTATTGAACGTAGCTGGGAAGCAGTTGTT<br>GTTGCAATGCTGGATGCAGTTCATAGTCCGCAGAGCAAACAGTTTAGCGAAATTCTGATTTATCAG<br>GGTCTGGATGAAAGCGGTCTGATTCATAATCGTATGGTTCAGGCAAGCGATCGTTTTCTGCAGTAT<br>ATTCATCAGGTTCTGCGTCATGCAGTTACCCAGGGTGAACTGCCGATTAATCTGGATCTGCAGACC<br>AGCATTGGTGTTTTTAAAGGTCTGATTACCGGTCTGCTGTATGAAGGTCTGCGTAGCAAAGATCAG<br>CAGGCACAGATTATCAAAGTTGCACTGGGTAGCTTTTGGGCACTGCTGCGTGAACCGCCTCGTTTT<br>CTGCTGTGTGAAGAAGCACAGATTAAACAGGTGAAATCCTTCGAATAA
PhlF	Gene	ATGGCACGTACCCCGAGCCGTAGCAGCATTGGTAGCCTGCGTAGTCCGCATACCCATAAAGCAATT<br>CTGACCAGCACCATTGAAATCCTGAAAGAATGTGGTTATAGCGGTCTGAGCATTGAAAGCGTTGCA<br>CGTCGTGCCGGTGCAAGCAAACCGACCATTTATCGTTGGTGGACCAATAAAGCAGCACTGATTGCC<br>GAAGTGTATGAAAATGAAAGCGAACAGGTGCGTAAATTTCCGGATCTGGGTAGCTTTAAAGCCGAT<br>CTGGATTTTCTGCTGCGTAATCTGTGGAAAGTTTGGCGTGAAACCATTTGTGGTGAAGCATTTCGT<br>TGTGTTATTGCAGAAGCACAGCTGGACCCTGCAACCCTGACCCAGCTGAAAGATCAGTTTATGGAA<br>CGTCGTCGTGAGATGCCGAAAAAACTGGTTGAAAATGCCATTAGCAATGGTGAACTGCCGAAAGAT<br>ACCAATCGTGAACTGCTGCTGGATATGATTTTTGGTTTTTGTTGGTATCGCCTGCTGACCGAACAG<br>CTGACCGTTGAACAGGATATTGAAGAATTTACCTTCCTGCTGATTAATGGTGTTTGTCCGGGTACA<br>CAGCGTTAA
YFP	Gene	ATGGTGAGCAAGGGCGAGGAGCTGTTCACCGGGGTGGTGCCCATCCTGGTCGAGCTGGACGGCGAC<br>GTAAACGGCCACAAGTTCAGCGTGTCCGGCGAGGGCGAGGGCGATGCCACCTACGGCAAGCTGACC<br>CTGAAGTTCATCTGCACCACCGGCAAGCTGCCCGTGCCCTGGCCCACCCTCGTGACCACCTTCGGC<br>TACGGCCTGCAATGCTTCGCCCGCTACCCCGACCACATGAAGCTGCACGACTTCTTCAAGTCCGCC<br>ATGCCCGAAGGCTACGTCCAGGAGCGCACCATCTTCTTCAAGGACGACGGCAACTACAAGACCCGC<br>GCCGAGGTGAAGTTCGAGGGCGACACCCTGGTGAACCGCATCGAGCTGAAGGGCATCGACTTCAAG<br>GAGGACGGCAACATCCTGGGGCACAAGCTGGAGTACAACTACAACAGCCACAACGTCTATATCATG<br>GCCGACAAGCAGAAGAACGGCATCAAGGTGAACTTCAAGATCCGCCACAACATCGAGGACGGCAGC<br>GTGCAGCTCGCCGACCACTACCAGCAGAACACCCCCATCGGCGACGGCCCCGTGCTGCTGCCCGAC<br>AACCACTACCTGAGCTACCAGTCCGCCCTGAGCAAAGACCCCAACGAGAAGCGCGATCACATGGTC<br>CTGCTGGAGTTCGTGACCGCCGCCGGGATCACTCTCGGCATGGACGAGCTGTACAAGTAA
TetR	Gene	ATGTCCAGATTAGATAAAAGTAAAGTGATTAACAGCGCATTAGAGCTGCTTAATGAGGTCGGAATC<br>GAAGGTTTAACAACCCGTAAACTCGCCCAGAAGCTAGGTGTAGAGCAGCCTACATTGTATTGGCAT<br>GTAAAAAATAAGCGGGCTTTGCTCGACGCCTTAGCCATTGAGATGTTAGATAGGCACCATACTCAC<br>TTTTGCCCTTTAGAAGGGGAAAGCTGGCAAGATTTTTTACGTAATAACGCTAAAAGTTTTAGATGT

		GCTTTACTAAGTCATCGCGATGGAGCAAAAGTACATTTAGGTACACGGCCTACAGAAAAACAGTAT<br>GAAACTCTCGAAAATCAATTAGCCTTTTTATGCCAACAAGGTTTTTCACTAGAGAATGCATTATAT<br>GCACTCAGCGCTGTGGGGCATTTTACTTTAGGTTGCGTATTGGAAGATCAAGAGCATCAAGTCGCT<br>AAAGAAGAAAGGGAAACACCTACTACTGATAGTATGCCGCCATTATTACGACAAGCTATCGAATTA<br>TTTGATCACCAAGGTGCAGAGCCAGCCTTCTTATTCGGCCTTGAATTGATCATATGCGGATTAGAA<br>AAACAACTTAAATGTGAAAGTGGGTCCTAA
LacI	Gene	ATGAAACCAGTAACGTTATACGATGTCGCAGAGTATGCCGGTGTCTCTTATCAGACCGTTTCCCGC<br>GTGGTGAACCAGGCCAGCCACGTTTCTGCGAAAACGCGGGAAAAAGTGGAAGCGGCGATGGCGGAG<br>CTGAATTACATTCCCAACCGCGTGGCACAACAACTGGCGGGCAAACAGTCGTTGCTGATTGGCGTT<br>GCCACCTCCAGTCTGGCCCTGCACGCGCCGTCGCAAATTGTCGCGGCGATTAAATCTCGCGCCGAT<br>CAACTGGGTGCCAGCGTGGTGGTGTCGATGGTAGAACGAAGCGGCGTCGAAGCCTGTAAAGCGGCG<br>GTGCACAATCTTCTCGCGCAACGCGTCAGTGGGCTGATCATTAACTATCCGCTGGATGACCAGGAT<br>GCCATTGCTGTGGAAGCTGCCTGCACTAATGTTCCGGCGTTATTTCTTGATGTCTCTGACCAGACA<br>CCCATCAACAGTATTATTTTCTCCCATGAGGACGGTACGCGACTGGGCGTGGAGCATCTGGTCGCA<br>TTGGGTCACCAGCAAATCGCGCTGTTAGCGGGCCCATTAAGTTCTGTCTCGGCGCGTCTGCGTCTG<br>GCTGGCTGGCATAAATATCTCACTCGCAATCAAATTCAGCCGATAGCGGAACGGGAAGGCGACTGG<br>AGTGCCATGTCCGGTTTTCAACAAACCATGCAAATGCTGAATGAGGGCATCGTTCCCACTGCGATG<br>CTGGTTGCCAACGATCAGATGGCGCTGGGCGCAATGCGCGCCATTACCGAGTCCGGGCTGCGCGTT<br>GGTGCGGATATCTCGGTAGTGGGATACGACGATACCGAAGATAGCTCATGTTATATCCCGCCGTTA<br>ACCACCATCAAACAGGATTTTCGCCTGCTGGGGCAAACCAGCGTGGACCGCTTGCTGCAACTCTCT<br>CAGGGCCAGGCGGTGAAGGGCAATCAGCTGTTGCCAGTCTCACTGGTGAAAAGAAAAACCACCCTG<br>GCGCCCAATACGCAAACCGCCTCTCCCCGCGCGTTGGCCGATTCATTAATGCAGCTGGCACGACAG<br>GTTTCCCGACTGGAAAGCGGGCAGTGA
AraC	Gene	ATGGCTGAAGCGCAAAATGATCCCCTGCTGCCGGGATACTCGTTTAATGCCCATCTGGTGGCGGGT<br>TTAACGCCGATTGAGGCCAACGGTTATCTCGATTTTTTTATCGACCGACCGCTGGGAATGAAAGGT<br>TATATTCTCAATCTCACCATTCGCGGTCAGGGGGTGGTGAAAAATCAGGGACGAGAATTTGTTTGC<br>CGACCGGGTGATATTTTGCTGTTCCCGCCAGGAGAGATTCATCACTACGGTCGTCATCCGGAGGCT<br>CGCGAATGGTATCACCAGTGGGTTTACTTTCGTCCGCGCGCCTACTGGCATGAATGGCTTAACTGG<br>CCGTCAATATTTGCCAATACGGGGTTCTTTCGCCCGGATGAAGCGCACCAGCCGCATTTCAGCGAC<br>CTGTTTGGGCAAATCATTAACGCCGGGCAAGGGGAAGGGCGCTATTCGGAGCTGCTGGCGATAAAT<br>CTGCTTGAGCAATTGTTACTGCGGCGCATGGAAGCGATTAACGAGTCGCTCCATCCACCGATGGAT<br>AATCGGGTACGCGAGGCTTGTCAGTACATCAGCGATCACCTGGCAGACAGCAATTTTGATATCGCC<br>AGCGTCGCACAGCATGTTTGCTTGTCGCCGTCGCGTCTGTCACATCTTTTCCGCCAGCAGTTAGGG<br>ATTAGCGTCTTAAGCTGGCGCGAGGACCAACGTATCAGCCAGGCGAAGCTGCTTTTGAGCACCACC<br>CGGATGCCTATCGCCACCGTCGGTCGCAATGTTGGTTTTGACGATCAACTCTATTTCTCGCGGGTA<br>TTTAAAAAATGCACCGGGGCCAGCCCGAGCGAGTTCCGTGCCGGTTGTGAAGAAAAAGTGAATGAT<br>GTAGCCGTCAAGTTGTCATAA
P_{Tac}	Promoter	TGTTGACAATTAATCATCGGCTCGTATAATGTGTGGAATTGTGAGCGCTCACAATT
P_{Tet}	Promoter	TTTTTTCCCTATCAGTGATAGAGATTGACATCCCTATCAGTGATAGAGATAATGAGCAC
P_{BAD}	Promoter	ACTTTTCATACTCCCGCCATTCAGAGAAGAAACCAATTGTCCATATTGCATCAGACATTGCCGTCA<br>CTGCGTCTTTTACTGGCTCTTCTCGCTAACCAAACCGGTAACCCCGCTTATTAAAAGCATTCTGTA<br>ACAAAGCGGGACCAAAGCCATGACAAAAACGCGTAACAAAAGTGTCTATAATCACGGCAGAAAAGT<br>CCACATTGATTATTTGCACGGCGTCACACTTTGCTATGCCATAGCATTTTTATCCATAAGATTAGC<br>GGATCCTACCTGACGCTTTTTATCGCAACTCTCTACTGTTTCTCCATACCCGTTTTTTTGGGCTAG<br>C
P_{BM3R1}	Promoter	TCTGATTCGTTACCAATTGACGGAATGAACGTTCATTCCGATAATGCTAGC
P_{AmtR}	Promoter	GATTCGTTACCAATTGACAGTTTCTATCGATCTATAGATAATGCTAGC
P_{SrpR}	Promoter	GATTCGTTACCAATTGACAGCTAGCTCAGTCCTAGGTATATACATACATGCTTGTTTGTTTGTAAA<br>C
P_{LitR}	Promoter	GATTCGTTACCAATTGACAAATTTATAAATTGTCAGTATAATGCTAGC
P_{PhlF}	Promoter	TCTGATTCGTTACCAATTGACATGATACGAAACGTACCGTATCGTTAAGGT
BydvJ	Ribozyme	AGGGTGTCTCAAGGTGCGTACCTTGACTGATGAGTCCGAAAGGACGAAACACCCCTCTACAAATAA<br>TTTTGTTTAA
PlmJ	Ribozyme	AGTCATAAGTCTGGGCTAAGCCCACTGATGAGTCGCTGAAATGCGACGAAACTTATGACCTCTACA<br>AATAATTTTGTTTAA
SarJ	Ribozyme	AGACTGTCGCCGGATGTGTATCCGACCTGACGATGGCCCAAAAGGGCCGAAACAGTCCTCTACAAA<br>TAATTTTGTTTAA
RiboJ10	Ribozyme	AGCGCTCAACGGGTGTGCTTCCCGTTCTGATGAGTCCGTGAGGACGAAAGCGCCTCTACAAATAAT<br>TTTGTTTAA

RiboJ53	Ribozyme	AGCGGTCAACGCATGTGCTTTGCGTTCTGATGAGACAGTGATGTCGAAACCGCCTCTACAAATAATTTTGTTTAA
RiboJ	Ribozyme	AGCTGTCACCGGATGTGCTTTCCGGTCTGATGAGTCCGTGAGGACGAAACAGCCTCTACAAATAATTTTGTTTAA
L3S2P55	Terminator	CTCGGTACCAAAGACGAACAATAAGACGCTGAAAAGCGTCTTTTTTCGTTTTGGTCC
L3S2P24	Terminator	CTCGGTACCAAATTCCAGAAAAGACACCCGAAAGGGTGTTTTTTCGTTTTGGTCC
L3S2P11	Terminator	CTCGGTACCAAATTCCAGAAAAGAGACGCTTTCGAGCGTCTTTTTTCGTTTTGGTCC
ECK120029600	Terminator	TTCAGCCAAAAAACTTAAGACCGCCGGTCTTGTCCACTACCTTGCAGTAATGCGGTGGACAGGATCGGCGGTTTTCTTTTCTCTTCTCAA
ECK120033737	Terminator	GGAAACACAGAAAAAAGCCCGCACCTGACAGTGCGGGCTTTTTTTTTCGACCAAAGG
L3S2P21	Terminator	CTCGGTACCAAATTCCAGAAAAGAGGCCTCCCGAAAGGGGGGCCTTTTTTCGTTTTGGTCC
BT1[a]	Terminator (Bidirectional)	AAAGCCCCCGGAAGATCACCTTCCGGGGGCTTTTTTATTGCGC**CCAAAAGTAAAAACCCGCCGAAGCGGGTTTTTACGTAAAACAGGTGAAACT**

a. Forward terminator (ECK120033736) is underlined, and reverse terminator (ECK120010818) is in bold.

Appendix References

Klumpp S, Zhang Z, Hwa T (2009) Growth rate-dependent global effects on gene expression in bacteria. *Cell* **139:** 1366-1375

Nielsen AAK, Der B, Shin J, Vaidyanathan P, Paralanov V, Strychalski EA, Ross D, Densmore D, Voigt CA (2016) Genetic circuit design automation. *Science* **352:** aac7341

Roberts A, Trapnell C, Donaghey J, Rinn LJ, Pachter L (2011) Improving RNA-Seq expression estimates by correcting for fragment bias. *Genome biology* **12:** R22

Robinson MD, Oshlack A (2010) A scaling normalization method for differential expression analysis of RNA-seq data. *Genome biology* **11:** R25
